# Obinutuzumab versus rituximab-based chemotherapy in high-tumor burden indolent B-cell lymphoma: a real-world comparative study

**DOI:** 10.3389/fimmu.2026.1857350

**Published:** 2026-06-18

**Authors:** Xin Wan, Dechuan Liu, Haotian Wang, Wei Guo, Xingtong Wang, Yangzhi Zhao, Ou Bai

**Affiliations:** 1 Department of Hematology, The First Hospital of Jilin University, Changchun, Jilin, China; 2 Department of Donation Intensive Care Unit, The First Hospital of Jilin University, Changchun, Jilin, China

**Keywords:** adverse events, B-iNHL, obinutuzumab, real-world data, rituximab

## Abstract

**Background:**

Patients with previously untreated follicular lymphoma (FL) or marginal zone lymphoma (MZL) with high-tumor burden represent a subset with unfavorable prognosis. However, the efficacy of obinutuzumab in this specific high-risk population remains incompletely characterized. This study aimed to compare the efficacy and safety of obinutuzumab-based versus rituximab-based chemotherapy in patients with high-risk features of FL and MZL.

**Methods:**

A retrospective analysis was conducted on 186 patients with histologically confirmed FL or MZL who received either obinutuzumab-based (n=92) or rituximab-based (n=94) chemotherapy at the First Hospital of Jilin University from February 2016 to February 2025. A propensity score overlap weight (PSOW) analysis was performed to adjust statistical influences between the two groups. Efficacy evaluation included complete response rate (CRR), objective response rate (ORR), progression-free survival (PFS), overall survival (OS), and progression of disease within 24 months (POD24).

**Results:**

After induction therapy, the CRR was significantly higher in the obinutuzumab group compared to the rituximab group (82.6% vs. 54.3%, p=0.014). With a median follow-up of 31.5 months, the obinutuzumab group demonstrated superior 3-year PFS (81.4% vs. 62.1%, p=0.0026) and 3-year OS (99.0% vs. 87.3%, p=0.004). The incidence of POD24 was lower in the obinutuzumab group (13.0% vs. 24.5%, p=0.046). Multivariable analysis identified rituximab-based treatment as an independent risk factor for inferior OS (HR 9.6, p=0.026). Safety profiles were similar between the two groups, with no significant differences in adverse event rates.

**Conclusion:**

Obinutuzumab-based chemotherapy was associated with significantly higher CRR, improved survival outcomes, and a lower POD24 rate compared to rituximab-based chemotherapy in patients with high-tumor burden of FL and MZL. These findings support the preferential use of obinutuzumab in this high-risk population.

## 1 Introduction

Indolent B-cell non-Hodgkin lymphoma (B-iNHL) represents a heterogeneous group of malignancies characterized by an indolent clinical course but a persistent risk of disease progression and transformation (1–3). Among these entities, follicular lymphoma grade 1-3a (FL 1-3a) and marginal zone lymphoma (MZL) are the most prevalent subtypes, collectively accounting for approximately 30% of all non-Hodgkin lymphomas (4). While both diseases are generally associated with prolonged survival, a subset of patients presenting with high tumor burden exhibits significantly worse outcomes, including shorter time to progression and inferior overall survival (5).

The Groupe d’Etude des Lymphomes Folliculaires (GELF) criteria have been widely adopted to define high tumor burden in FL, guiding the initiation of systemic therapy (6). Although no universally accepted high tumor burden criteria have been established specifically for MZL, the GELF framework has been increasingly referenced in clinical practice and research settings to stratify patients with advanced-stage MZL who may benefit from chemoimmunotherapy over watchful waiting, serving as a pragmatic surrogate for treatment intervention (7, 8).

Rituximab, a type I anti-CD20 monoclonal antibody, in combination with chemotherapy, has been the cornerstone of treatment for high-tumor-burden B-iNHL for over two decades (9). More recently, obinutuzumab, a novel humanized glyco-engineered type II anti-CD20 monoclonal antibody engineered for enhanced antibody-dependent cellular cytotoxicity and direct cell death induction, has demonstrated superior efficacy in the treatment of B-iNHL (10, 11). However, patients with high-tumor-burden were often underrepresented in these pivotal trials, and real-world data comparing the two agents specifically in this high-risk population remain limited.

This real-world study aims to compare the efficacy and safety of obinutuzumab-based versus rituximab-based chemotherapy in a cohort of patients with high-tumor burden of FL 1-3a and MZL, employing rigorous statistical methods to mitigate temporal biases and provide clinically meaningful evidence for treatment selection in this challenging population.

## 2 Methods

### 2.1 Study design and patient population

**Figure 1:**
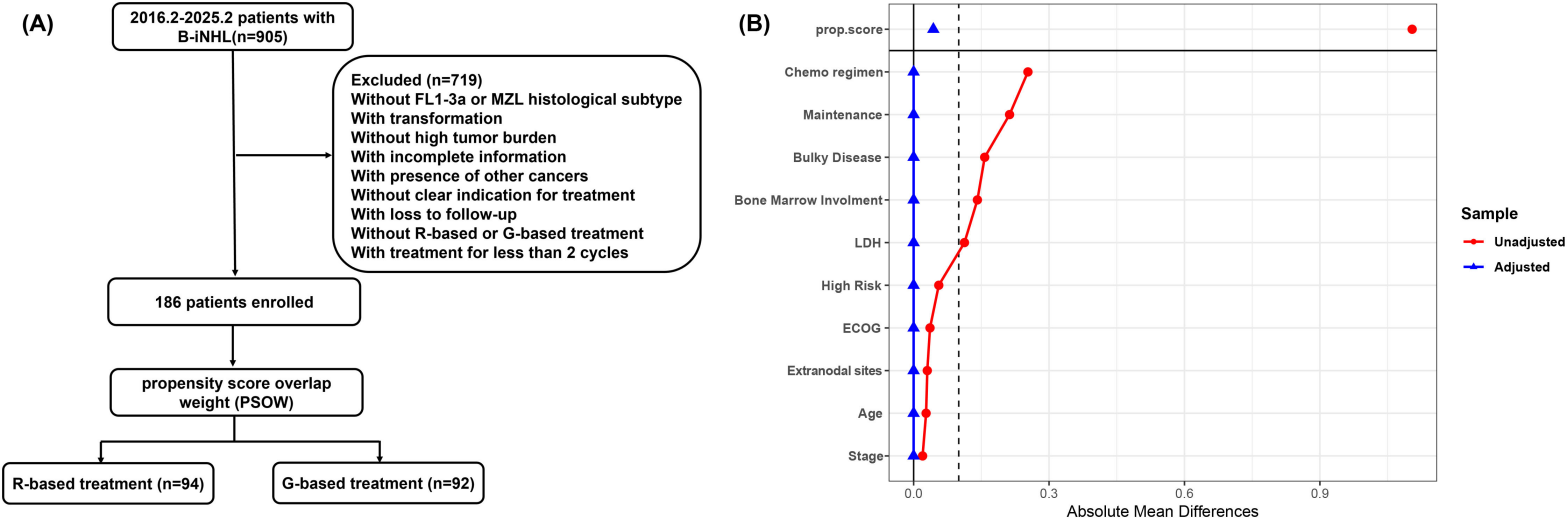
**(A)** Study flowchart. **(B)** Assessing balance before and after propensity score-overlap weighting.

This retrospective, single-center study was conducted at the First Hospital of Jilin University. Consecutive patients with FL grade 1-3a and MZL who received first-line or relapsed/refractory treatment with obinutuzumab- or rituximab-based immunochemotherapy between February 2016 and February 2025 were screened for eligibility. As illustrated in **Figure 1**, 186 patients were ultimately enrolled in this study. This study adhered to the principles of the Declaration of Helsinki and was approved by the Institutional Review Board of Jilin University. Informed consent was obtained from all patients. The data cutoff date for the final analysis was March 2026.

### 2.2 Inclusion and exclusion criteria

Patients were included if they met the following criteria: (A) histologically confirmed diagnosis of FL grade 1-3a and MZL, according to the 2016 or 2022 World Health Organization classification of hematological malignancies; (B) age ≥18 years; (C) with high tumor burden disease according to the GELF criteria (6); (D) receipt of obinutuzumab- or rituximab-based chemotherapy. Patients were excluded if they had: (A) histologically confirmed transformation to aggressive lymphoma; (B) concurrent secondary malignancies; (C) incomplete clinical records; (D) severe organ dysfunction unrelated to lymphoma.

### 2.3 Treatment regimens and assessments

All patients received chemotherapy prior to the initiation of obinutuzumab or rituximab therapy in cycle 1. The administration schedule of anti-CD20 monoclonal antibody was as follows: obinutuzumab was administered as an intravenous infusion of 100 mg on day 1, 900 mg on day 2, 1000 mg on days 8 and 15 of cycle 1, and 1000 mg on day 1 of subsequent cycles; rituximab was administered as an intravenous infusion of 375 mg/m² on day 1 of each cycle. Chemotherapy backbone selection was tailored to clinical characteristics: bendamustine was preferentially used for patients presenting with hyperleukocytosis or splenomegaly, while CHOP/CDOP (cyclophosphamide, doxorubicin, vincristine, prednisone/cyclophosphamide, pegylated liposomal doxorubicin, vincristine, and prednisone) or CVP (cyclophosphamide, vincristine, prednisone) regimens were selected for patients with nodal or extranodal involvement. This practice reflected real-world clinical decision-making and ensured that patients received appropriate therapy based on their dominant disease manifestations. Furthermore, patients with FL grade 1-3a were recommended to receive 24 months of the same CD20 monoclonal antibody maintenance therapy after induction.

Response assessment was performed at mid-induction (cycle 5, day 1) and at the end of induction (after completion of 6 cycles or as clinically indicated) using the Lugano classification criteria (12). Objective response rate (ORR) was defined as the percentage of patients achieving complete response (CR) or partial response (PR). Progression-free survival (PFS) was defined as the time from treatment initiation to disease progression, relapse, or death from any cause. Overall survival (OS) was defined as the time from treatment initiation to death from any cause. Progression of disease within 24 months (POD24) was defined as disease progression or relapse within 24 months of treatment initiation. Adverse events were graded according to the Common Terminology Criteria for Adverse Events version 5.0. The primary endpoints included ORR, complete response rate (CRR), and 3-year PFS. The secondary endpoints were incidence of adverse events (AEs) and 3-year OS.

### 2.4 Statistical analysis

In retrospective analyses, baseline clinical characteristics often differ substantially between treatment groups, which may introduce considerable bias into effect estimates. To address this, propensity score overlap weighting (PSOW) was employed. Balance in baseline covariates was assessed using standardized mean differences (SMD), with a threshold of <0.1 indicating adequate balance. This method downweights patients with extreme propensity scores and upweights those with overlapping propensity distributions between groups, thereby approximating the covariate balance typically observed in randomized controlled trials (13–15). Baseline characteristics were compared between treatment groups using chi-square or Fisher’s exact tests for categorical variables. The Kaplan-Meier method was used to estimate PFS and OS, with differences between groups assessed by the log-rank test. Univariate and multivariate cox proportional hazards models were employed to identify prognostic factors for PFS and OS. Variables with p<0.1 in univariate analysis were entered into the multivariate model. Given the later availability of obinutuzumab in clinical practice (approved in China in August 2021), the two treatment cohorts are separated temporally, with the rituximab cohort treated predominantly from 2016 to 2021 and the obinutuzumab cohort from 2021 to 2025. To mitigate potential bias arising from differences in enrollment periods, follow-up duration was uniformly calculated from the enrollment date of the first patient in each group, with a common censoring point set at 50 months of follow-up for both groups. All statistical tests were two-sided, with p<0.05 considered statistically significant. Statistical analyses were performed using SPSS version 27.0 and R version 4.4.3.

## 3 Results

### 3.1 Patient baseline characteristics

A total of 186 patients met the inclusion criteria, comprising 92 patients in the obinutuzumab-based group (G-group) and 94 patients in the rituximab-based group (R-group). Baseline characteristics are summarized in **Table 1**. Among all patients, 184 (98.9%) presented with newly diagnosed disease, while 2 (1.1%) had relapsed/refractory disease. The median age at enrollment was 53 years (range: 29–84 years), with 96 males (51.6%) and 90 females (48.4%). The majority of patients (92.5%) had advanced-stage disease. B symptoms were present in 84 patients (45.2%), the eastern cooperative oncology group (ECOG) performance status >1 in 35 patients (18.8%), and bone marrow involvement was present in 106 patients (57.0%). Elevated lactate dehydrogenase (LDH) levels were observed in 49 patients (26.3%), and β2-microglobulin >3 mg/L was observed in 91 patients (48.9%). Other baseline characteristics, including extranodal organ involvement, were comparable between the two groups (**Table 1**). Compared with the rituximab group, the obinutuzumab group had a higher proportion of patients with mass ≥7cm (40.2% vs. 24.5%, p=0.022) and bone marrow involvement (64.1% vs. 50.0%, p=0.052). To minimize potential confounding arising from these imbalances and temporal trends in treatment selection, PSOW was applied. After OW adjustment, all baseline characteristics were well balanced between the two groups (**Table 1**).

**Table 1:** Baseline characteristics of 186 patients before and after propensity score-overlap weighting.

Clinical characteristics	Level	Before overlap weighting			After overlap weighting		
		G-group (n=92)	R-group (n=94)	P value	G-group (n=92)	R-group (n=94)	P value
Gender	Male	48 (52.2)	48 (51.1)	0.880	19.55	16.95	0.379
	Female	44(47.8)	46 (48.9)		15.68	18.28	
Age	≤65 >65	76 (82.6) 16 (17.4)	79 (84.0) 15 (16.0)	0.793	29.16 6.07	29.60 5.63	0.852
Diagnosis	FL 1-3a MZL	57 (62.0) 35 (38.0)	58 (61.7) 36 (38.3)	0.972	24.40 10.83	22.51 12.72	0.487
Clinical stage	I-II	6 (6.5)	8 (8.5)	0.607	2.49	2.49	1
	III-IV	86 (93.5)	86 (91.5)		32.7	32.7	
B symptom	Yes	43 (46.7)	41 (43.6)	0.529	15.59	12.30	0.217
	No	48 (52.2)	53 (56.4)		19.12	22.93	
ECOG	≤ 1	73 (79.3)	78 (83.0)	0.526	28.23	28.23	1
	>1	19 (20.7)	16 (17.0)		7.00	7.00	
IPI^*^	0~2	57 (62.0)	53 (56.4)	0.439	20.77	20.77	1
	≥3	35 (38.0)	41 (43.6)		14.46	14.46	
LDH	Normal	73 (79.3)	64 (68.1)	0.081	26.31	26.31	1
	Elevated	19 (20.7)	30 (31.9)		8.92	8.92	
β2MG >3mg/L	Yes	44 (47.8)	47 (50.0)	0.957	16.81	17.61	0.738
	No	47 (51.1)	46 (48.9)		18.35	17.17	
BM involvement	Yes	59 (64.1)	47 (50.0)	0.052	19.44	19.44	1
	No	33 (35.9)	47 (50.0)		15.79	15.79	
Mass ≥7cm	Yes	37 (40.2)	23 (24.5)	0.022	11.29	11.29	1
	No	55 (59.8)	71 (75.5)		23.94	23.94	
Extranodal sites≥2	Yes	39 (42.4)	37 (39.4)	0.674	13.80	13.80	1
	No	53 (57.6)	57 (60.6)		21.43	21.43	
Chemotherapy regimens	Bendamustine	37 (40.2)	14 (14.9)	<0.0001	9.67	9.67	1
	No-Bendamustine	55 (59.8)	80 (85.2)		25.56	25.56	
Maintenance therapy	Yes	45 (48.9)	26 (27.7)	0.003	14.73	14.73	1
	No	47 (51.1)	68 (72.3)		20.50	20.50	

FL 1-3a, follicular lymphoma grade 1-3a; MZL, marginal zone lymphoma; G, obinutuzumab; R, rituximab; β2MG, beta-2 microglobulin; BM, bone marrow; ECOG, the eastern cooperative oncology group; IPI, international prognostic index; LDH, lactate dehydrogenase. ^*^For patients with FL grade 1-3a, the Follicular Lymphoma International Prognostic Index (FLIPI) was applied; for those with MZL, the MZL International Prognostic Index (MZL-IPI) was used.

### 3.2 Efficacy outcomes

At the first comprehensive evaluation (cycle 5, day 1), 116 patients (62.4%) achieved CR, with an ORR of 94.6%. At the end of induction therapy, the ORR and CRR were 93.5% and 68.3%, respectively (**Table 2**). Subgroup analysis by treatment regimen revealed marked differences in response rates. After four treatment cycles, the CRR was significantly higher in the obinutuzumab group compared with the rituximab group (72.8% vs. 52.1%, p=0.033). At the end of induction, this difference persisted, with CRR of 82.6% in the obinutuzumab group versus 54.3% in the rituximab group (p=0.014, **Table 2**). These findings collectively indicate that obinutuzumab can rapidly alleviate the high tumor burden state in patients with B-iNHL.

**Table 2:** Short-term efficacy of different induction therapy regimens.

Response	n (%)	G-group	R-group	P value^#^
First comprehensive evaluation (cycle 5, day 1)				
OR	176 (94.6)	91 (98.9)	85 (90.4)	0.033
CR	116 (62.4)	67 (72.8)	49 (52.1)	
PR	60 (32.3)	24 (26.1)	36 (38.3)	
PD	10 (5.4)	1 (1.1)	9 (9.6)	
At the end of induction therapy				
OR	174 (93.5)	89 (96.7)	85 (90.4)	0.014
CR	127 (68.3)	76 (82.6)	51 (54.3)	
PR	47 (25.3)	13 (14.1)	34 (36.2)	
PD	12 (6.5)	3 (3.3)	9 (9.6)	

G, obinutuzumab; R, rituximab; OR, objective response; CR, complete response; PR, partial response; SD, stable disease; PD, progressive disease. ^#^P value after propensity score-overlap weighting.

### 3.3 Survival outcomes

**Figure 2:**
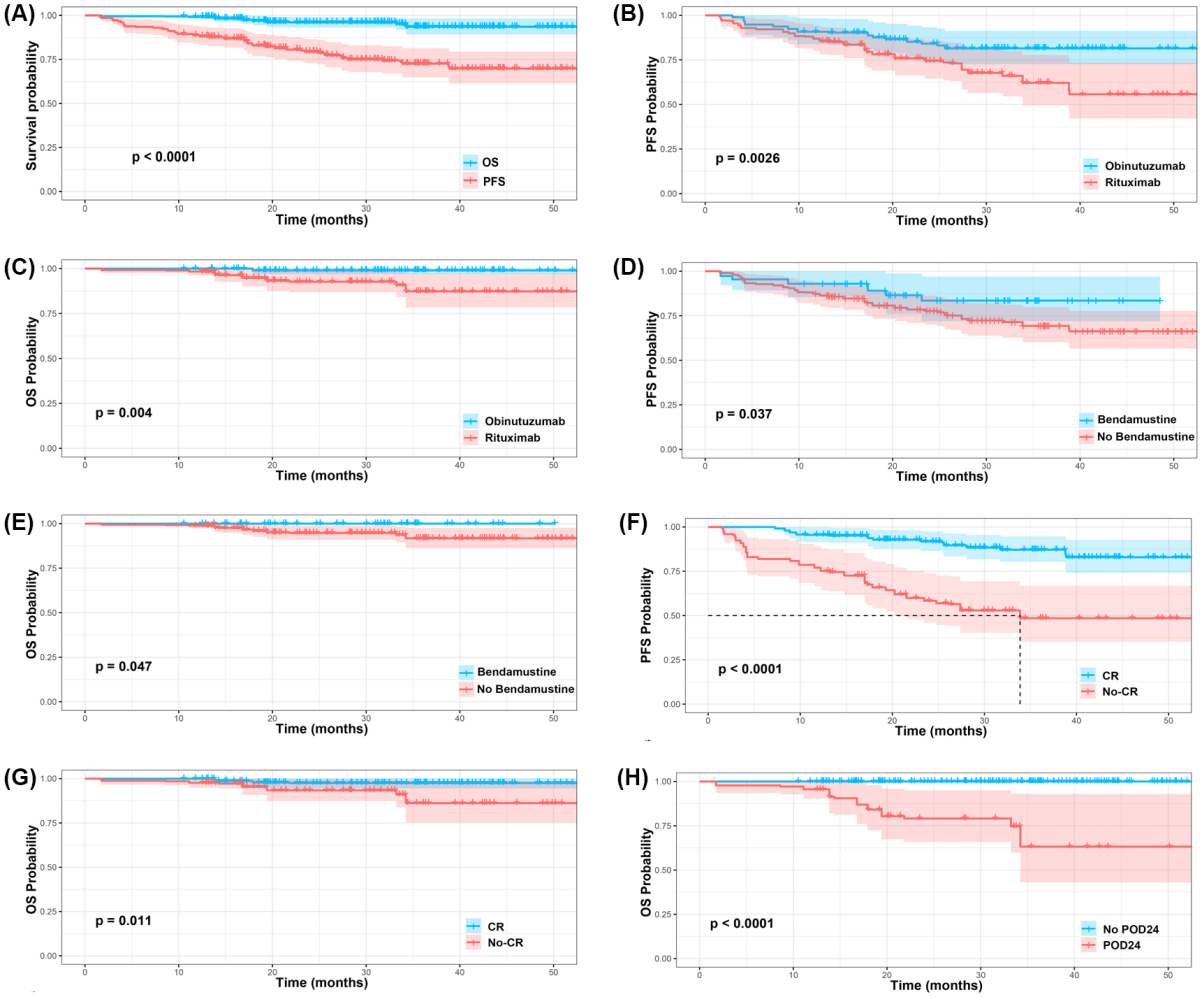
Overlap weighting-adjusted Kaplan–Meier curves of survival. **(A)** Kaplan-Meier curve for progression-free survival (PFS) and overall survival (OS) evaluation in overall 186 patients. **(B)** Kaplan-Meier curve for PFS evaluation of patients receiving obinutuzumab-based vs. rituximab-based therapy. **(C)** Kaplan-Meier curve for OS evaluation in patients receiving obinutuzumab-based vs. rituximab-based therapy. **(D)** Kaplan-Meier curve for PFS evaluation of patients receiving bendamustine-based vs. no bendamustine-based therapy. **(E)** Kaplan-Meier curve for OS evaluation in patients receiving bendamustine-based vs. no bendamustine-based therapy. **(F)** Kaplan-Meier curve for PFS evaluation in patients achieving complete remission (CR) vs. not achieving CR after 4 cycles therapy. **(G)** Kaplan-Meier curve for OS evaluation in patients achieving CR vs. not achieving CR after 4 cycles therapy. **(H)** Kaplan-Meier curve for OS of POD24 vs. non-POD24.

With a median follow-up of 31.5 months for the total population (32 months for the G-group and 30 months for the R-group), neither median PFS nor median OS was reached in the overall cohort. The 3-year PFS rate was 72.6%, while the 3-year OS rate was 93.6% (**Figure 2A**). Patients treated with obinutuzumab-based regimens demonstrated significantly superior survival outcomes compared to those receiving rituximab-based therapy. The 3-year PFS rate was 81.4% in the G-group versus 62.1% in the R-group (p=0.0026) (**Figure 2B**). The 3-year OS rate was 99.0% in the G-group versus 87.3% in the R-group (p=0.004) (**Figure 2C**).

Patients treated with bendamustine-based regimens demonstrated significantly superior survival compared with those receiving other therapies, with 3-year PFS rate of 83.5% versus 69.3% (p=0.037) and 3-year OS rate of 100% versus 91.7% (p=0.047) (**Figures 2D, E**). Response depth emerged as another critical determinant of long-term prognosis. Patients who achieved CR after 4 cycles of induction therapy had markedly better outcomes than non-CR patients, with 3-year PFS rates of 87.2% versus 48.5% (p<0.0001) and 3-year OS rates of 97.5% versus 86.2% (p=0.011) (**Figures 2F, G**). These findings underscore the importance of early deep remission in sustaining durable disease control. Overall, 35 patients (18.8%) experienced POD24. The incidence of POD24 was significantly lower in the obinutuzumab group compared with the rituximab group (13.0% [12/92] vs. 24.5% [23/94], p=0.046). Survival analysis demonstrated that patients with POD24 had a significantly inferior 3-year OS compared to those without POD24 (63.2% vs. 100%, p<0.0001) (**Figure 2H**), confirming the prognostic significance of this endpoint.

### 3.4 Prognostic factor analysis

Univariate analysis identified the following factors as significantly associated with inferior PFS: male, high risk, elevated LDH, β2-microglobulin >3 mg/L, bone marrow involvement, failure to achieve CR after 4 cycles, and rituximab-based treatment. Factors associated with inferior OS included bone marrow involvement, and rituximab-based treatment (**Table 3**). Multivariate cox regression analysis demonstrated that male (HR 2.186, 95% CI 1.118-4.273, p=0.022), high risk (HR 2.073, 95% CI 1.068-4.024, p=0.031), and failure to achieve CR after 4 cycles (HR 4.309, 95% CI 2.042-9.091, p=0.0001) were independent prognostic factors for PFS (**Table 4**), while rituximab-based treatment (HR 9.6, 95% CI 1.318-69.930, p=0.026) was an independent prognostic factor for OS (**Table 5**).

**Table 3:** Univariate analyses of PFS and OS.

Characteristics	Overall	PFS		OS	
		HR (95% CI)	P value	HR (95% CI)	P value^#^
Male	96 (51.6)	2.083 (1.034-4.195)	0.039	5.989 (0.752-47.7)	0.091
Age>65	31 (16.7)	0.527 (0.184-1.511)	0.233	2.609 (0.684-9.953)	0.16
Advanced stage	172 (92.5)	4.268 (0.583-31.24)	0.153	27066448^&^	<0.0001
B symptom	84 (45.2)	1.13 (0.588-2.172)	0.714	1.155 (0.323-4.135)	0.825
ECOG >1	35 (18.8)	1.845 (0.718-4.74)	0.203	1.339 (0.289-6.201)	0.709
High Risk^*^	76 (40.9)	2.018 (1.045-3.895)	0.037	3.075 (0.325-0.774)	0.11
Elevated LDH	49 (26.3)	2.0 (1.004-3.985)	0.0487	2.782 (0.695-11.13)	0.148
β2MG >3mg/L	91 (48.9)	2.321 (1.176-4.582)	0.0152	3.477 (0.771-15.680)	0.105
BM involvement	106 (57.0)	2.241 (1.083-4.637)	0.0296	4.415 (1.154-16.890)	0.03
Extranodal sites≥2	76 (40.9)	1.933 (0.998-3.744)	0.051	0.781 (0.191-3.186)	0.718
Mass ≥7cm	60 (32.3)	1.327 (0.659-2.671)	0.428	0.598 (0.115-3.118)	0.541
R-based therapy	94 (50.5)	2.149 (1.058 -4.365)	0.034	10.46 (1.307-83.650)	0.027
Maintenance therapy	71 (38.2)	0.9176 (0.471-1.789)	0.801	0.954 (0.235-3.869)	0.948
Chemotherapy regimens*	135 (72.6)	1.906 (0.766-4.744)	0.166	279586763^&^	<0.0001
No CR after 4 cycles	70 (37.6)	5.0 (2.533-9.873)	<0.0001	4.308 (0.933-19.88)	0.061

PFS, progression-free survival; OS, overall survival; β2MG, beta-2 microglobulin; BM, bone marrow; ECOG, the eastern cooperative oncology group; IPI, international prognostic index; LDH, lactate dehydrogenase; R, rituximab; CR, complete response. ^#^P value after propensity score-overlap weighting. ^*^For patients with FL grade 1-3a, the Follicular Lymphoma International Prognostic Index (FLIPI) was applied; for those with MZL, the MZL International Prognostic Index (MZL-IPI) was used. ^&^The HR value is too high, considering to be a false positive.

**Table 4:** Multivariate analyses of PFS.

Characteristics	PFS		
	HR	95% CI	P value^#^
Male	2.186	1.118-4.273	0.022
High_Risk^*^	2.073	1.068-4.024	0.031
Elevated LDH	0.802	0.340-1.893	0.615
β2MG >3mg/L	1.446	0.669-3.123	0.348
BM involvement	1.501	0.672-3.357	0.322
Extranodal sites≥2	1.639	0.787-3.416	0.187
No CR after 4 cycles	4.309	2.042-9.091	0.0001
R-based therapy	1.456	0.666-3.186	0.347

PFS, progression-free survival; LDH, lactate dehydrogenase; β2MG, beta-2 microglobulin; CR, complete response; R, rituximab. ^#^P value after propensity score-overlap weighting. ^*^For patients with FL grade 1-3a, the Follicular Lymphoma International Prognostic Index (FLIPI) was applied; for those with MZL, the MZL International Prognostic Index (MZL-IPI) was used.

**Table 5:** Multivariate analyses of OS.

Characteristics	OS		
	HR	95% CI	P value^#^
Male	6.734	0.841-53.910	0.072
BM involvement	3.967	0.948-16.610	0.059
No CR after 4 cycles	2.151	0.473-9.790	0.322
R-based therapy	9.600	1.318-69.930	0.026

OS, overall survival; CR, complete response; R, rituximab; ^#^P value after propensity score-overlap weighting.

### 3.5 Subgroup analyses

#### 3.5.1 Follicular lymphoma grade 1-3a subgroup (n=115)

Among the 115 patients with FL grade 1-3a, the median age was 49 years (range: 29–84 years). Advanced-stage disease was present in 94.8% (109/115), B symptoms in 40.0% (46/115), ECOG >1 in 10.4% (12/115), elevated LDH in 28.7% (33/115), and β2-microglobulin >3 mg/L in 40.9% (47/115). High-risk FLIPI (score ≥3) and FLIPI-2 (score ≥3) classifications were observed in 53.9% (62/115) and 41.7% (48/115) of patients, respectively (**Table 6**).

**Table 6:** Clinical characteristics of different pathological subtype.

Clinical characteristics	FL 1-3a (n=115) (%)	MZL (n=71) (%)
Male	57 (49.6)	39 (54.9)
Age>65	12 (10.4)	19 (26.8)
Advanced Stage	109 (94.8)	63 (88.7)
B symptom	46 (40.0)	38 (53.5)
ECOG >1	12 (10.4)	23 (32.4)
High risk^*^	62 (53.9)	14 (19.7)
LDH Elevated	33 (28.7)	16 (22.5)
β2MG >3mg/L	47 (40.9)	44 (62.0)
BM involvement	63 (54.8)	43 (60.6)
Mass ≥7cm	44 (38.3)	16 (22.5)
Extranodal sites≥2	46 (40.0)	30 (42.3)
POD24	24 (20.9)	11 (15.5)

FL 1-3a, follicular lymphoma grade 1-3a; MZL, marginal zone lymphoma; β2MG, beta-2 microglobulin; BM, bone marrow; ECOG, the eastern cooperative oncology group; LDH, lactate dehydrogenase; POD24, progression of disease within 24 months. ^*^For patients with FL grade 1-3a, the Follicular Lymphoma International Prognostic Index (FLIPI) was applied; for those with MZL, the MZL International Prognostic Index (MZL-IPI) was used.

**Figure 3:**
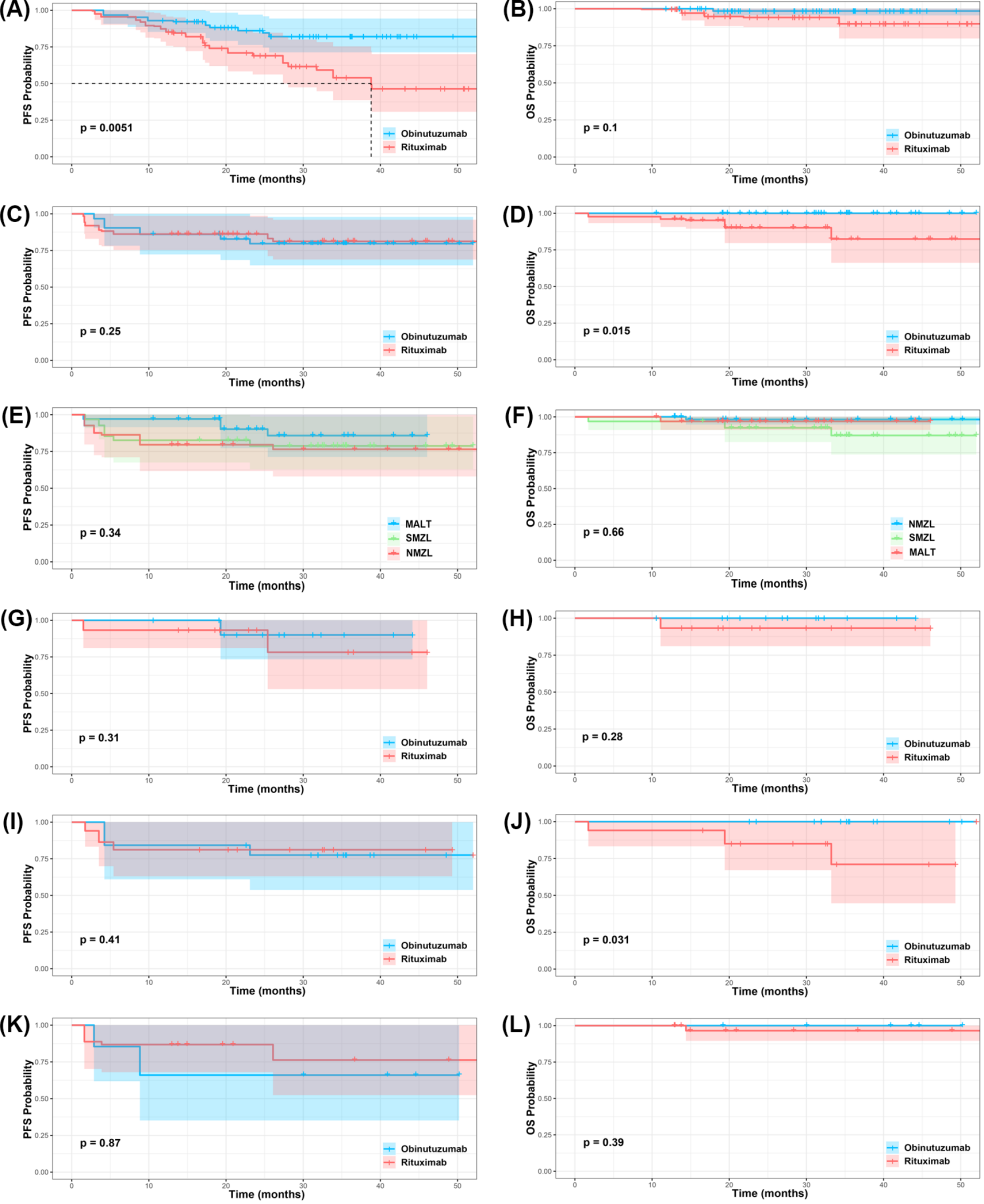
Overlap weighting-adjusted Kaplan–Meier curves of survival. **(A)** Kaplan-Meier curve for progression-free survival (PFS) evaluation in patients with follicular lymphoma grade 1-3a (FL 1-3a). **(B)** Kaplan-Meier curve for overall survival (OS) in patients with FL 1-3a. **(C)** Kaplan-Meier curve for PFS evaluation in patients with marginal zone lymphoma (MZL). **(D)** Kaplan-Meier curve for OS evaluation in patients with MZL. **(E)** Kaplan-Meier curve for PFS evaluation in patients among the three MZL subtypes. **(F)** Kaplan-Meier curve for OS evaluation in patients among the three MZL subtypes. **(G)** Kaplan-Meier curve for PFS evaluation in patients with MALT. **(H)** Kaplan-Meier curve for OS evaluation in patients with MALT. **(I)** Kaplan-Meier curve for PFS evaluation in patients with SMZL. **(J)** Kaplan-Meier curve for OS evaluation in patients with SMZL. **(K)** Kaplan-Meier curve for PFS evaluation in patients with NMZL. **(L)** Kaplan-Meier curve for OS evaluation in patients with NMZL.

With a median follow-up of 32 months, the overall ORR and CRR were 72.2% and 57.4%, respectively. Notably, patients receiving obinutuzumab-based therapy (n=57) achieved substantially higher response rates than those in the rituximab group (n=58), with an ORR of 84.2% versus 60.3% and a CRR of 71.9% versus 43.1% (p=0.005). This marked improvement in response depth translated into favorable long-term disease control, as evidenced by a significantly lower POD24 rate in the obinutuzumab group (12.3% vs. 29.3%, p=0.025). Consistent with these findings, obinutuzumab-based therapy conferred a significant PFS advantage, with 3-year PFS rates of 82.0% versus 54.0% (p=0.0051) (**Figure 3A**). Although a trend toward improved OS was observed with obinutuzumab (3-year OS: 98.5% vs. 89.8%), the difference did not reach statistical significance (p=0.1) (**Figure 3B**), likely reflecting the relatively short follow-up duration and the overall favorable prognosis of FL, which may limit the power to detect OS differences in this subgroup analysis.

#### 3.5.2 Marginal zone lymphoma subgroup (n=71)

The MZL subgroup included 26 patients with splenic MZL (SMZL), 27 with mucosa-associated lymphoid tissue (MALT) lymphoma, and 18 with nodal MZL (NMZL). The median age was 61 years (range: 39–82 years). Advanced-stage disease was present in 88.7% (63/71), B symptoms in 53.5% (38/71), ECOG >1 in 32.4% (23/71), elevated LDH in 22.5% (16/71), and β2-microglobulin >3 mg/L in 62% (44/71). The MZL-IPI score was intermediate or high risk in 85.9% (61/71), of which 14 patients (19.7%) were in the high-risk group (**Table 6**).

With a median follow-up of 31 months, the overall ORR and CR rate were 81.7% and 60.6%, respectively. By subtype, the ORR/CRR was 80.8%/57.7% for SMZL, 88.9%/70.4% for MALT, and 72.2%/50.0% for NMZL. Patients receiving obinutuzumab-based therapy (n=35) demonstrated superior outcomes compared with those receiving rituximab (n=36), with 3-year OS rates of 100% versus 82.4% (p=0.015) (**Figures 3C, D**). Notably, no significant differences in survival outcomes were observed among the three MZL subtypes (**Figures 3E, F**). By subtype, no significant differences in prognosis were observed between the obinutuzumab and rituximab groups among patients with MALT or NMZL; however, SMZL patients treated with obinutuzumab had significantly better outcomes than those treated with rituximab, with 3-year OS rates of 100% versus 71.0% (p=0.031) (**Figures 3G–L**).

### 3.6 Safety

The safety profile was comparable between the two treatment groups. The most common AEs of any grade were hematologic toxicities (65.2% vs. 59.6%), followed by infusion-related reactions (IRRs) (33.7% vs. 23.7%), renal function impairment (7.6% vs. 8.5%), liver function impairment (5.4% vs. 8.5%), respiratory system symptoms (5.4% vs. 3.2%), nausea (2.2% vs. 4.3%), and tumor lysis syndrome (TLS) (1.1% vs. 0%). Grade ≥3 AEs were predominantly hematologic (27.2% vs. 37.2%). No significant differences in the incidence of any-grade or grade ≥3 AEs were observed between the obinutuzumab and rituximab groups (all p>0.05) (**Table 7**). All toxicities were manageable and did not result in treatment discontinuation or treatment-related mortality.

**Table 7:** AEs in the treatment phase.

AE	G-group n (%)	R-group n (%)	P value
Any AE	74 (80.4)	81 (85.3)	
Hematologic	60 (65.2)	56 (59.6)	0.427
IRR	31 (33.7)	22 (23.7)	0.120
Renal function injury	7 (7.6)	8 (8.5)	0.821
Respiratory system	5 (5.4)	3 (3.2)	0.451
TLS	1 (1.1)	0 (0)	0.311
Liver function injury	5 (5.4)	8 (8.5)	0.411
Digestive system	2 (2.2)	4 (4.3)	0.422
Grade ≥ 3			
Hematologic	25 (27.2)	35 (37.2)	0.142
TLS	1 (1.1)	0 (0)	0.311

AE, adverse event; IRR, infusion-related reactions; TLS, tumor lysis syndrome.

## 4 Discussion

The term “B-iNHL” refers to a group of malignancies with similar disease courses and treatment strategies. However, the clinical outcomes of patients with high-tumor-burden FL and MZL remain suboptimal. Currently, immunochemotherapy remains the standard treatment for this patient population. In the treatment of high tumor burden FL, rituximab-based chemoimmunotherapy has historically served as the backbone of therapy. In a phase 2 single-arm study, patients with high tumor burden FL who received the R2-CHOP regimen achieved a CRR of 74% and an ORR of 94% at the end of the 6-cycle induction phase (16). In the phase 3 PRIMA trial, in which 68% of enrolled patients had high tumor burden, patients with FL 1-3a who had received initial R-CHOP treatment were randomly assigned to either 2 years of rituximab maintenance therapy or observation; with a median follow-up of 36 months, the 3-year PFS rate was 74.9% in the maintenance group, compared with 57.6% in the observation group (17). Furthermore, in the FOLL05 trial evaluating the initial treatment of patients with advanced-stage FL, the CRR was 73% among the 178 patients treated with R-CHOP (18). These landmark trials established rituximab-based therapy as the standard of care for high-tumor burden FL. However, comparable prospective data for MZL are limited, and the applicability of these outcomes to high-tumor-burden MZL remains uncertain given the lack of standardized high tumor burden criteria in this entity.

Additionally, previous randomized controlled trials have confirmed the therapeutic benefits of combining chemotherapy with the preferred CD20 antibody obinutuzumab. Obinutuzumab has demonstrated superior efficacy compared to rituximab, especially in patients with relapsed indolent NHL, and therapeutic responses have also been observed in rituximab-refractory patients (11, 19). In this real-world comparative study of 186 patients with high tumor burden FL 1-3a and MZL, obinutuzumab-based chemotherapy demonstrated significantly superior efficacy compared to rituximab-based regimens after induction therapy, with higher CR rates (82.6% vs. 54.3%, p=0.014), improved 3-year PFS (81.4% vs. 62.1%, p=0.0026), and enhanced 3-year OS (99.0% vs. 87.3%, p=0.004), indicating that the remissions achieved after induction therapy were sustained in most patients.

Several studies have been conducted to evaluate the efficacy of obinutuzumab in patients with high-risk B-iNHL. A phase 2 study investigating the combination of venetoclax, bendamustine, and obinutuzumab as first-line therapy for patients with high-risk FL showed a CRR of 73.2%, an ORR of 92.5%, and an estimated 2-year PFS rate of 87.5%; however, the safety of this regimen requires further investigation (20). The LYSA study (21), which evaluated the efficacy of obinutuzumab combined with lenalidomide in patients with FL 1-3a, high-tumor burden, and ECOG performance status ≤ 2, reported a CRR of 47% and an ORR of 92% at the end of induction; unfortunately, this study failed to meet its primary endpoint of CRR at the end of induction. Moreover, another phase 2 study evaluating obinutuzumab combined with lenalidomide as first-line therapy for high tumor burden FL 1-3a reported a 2-year PFS rate of 93.3% (22), which was slightly higher than that observed in our cohort. There are several potential reasons for this discrepancy. Firstly, the proportion of patients with ECOG ≥ 2 in our cohort was 18.8%, which was higher than that in previous studies (22, 23). Secondly, our study included a higher proportion of patients with advanced-stage disease (92.5%) and lymph nodes with a maximum diameter ≥7 cm (32.3%) compared to previous reports (23). These factors may contribute to the relatively shorter PFS observed in our cohort compared to earlier studies.

The high CR rates achieved in our obinutuzumab cohort (82.6%) warrant specific attention. Deep remission, particularly CRR, has been consistently associated with improved long-term outcomes in indolent lymphomas, including reduced risk of early progression and transformation (24). In our cohort, the markedly higher CRR with obinutuzumab likely contributes to the superior PFS and OS observed. Furthermore, the lower incidence of POD24 in the obinutuzumab group (13.0% vs. 24.5%) is clinically meaningful, as POD24 has emerged as a powerful surrogate for poor prognosis across various indolent lymphoma subtypes (8, 25).

The superior survival outcomes observed with bendamustine-based regimens in our cohort are consistent with findings from prospective randomized trials. In the phase 3 STiL trial, bendamustine plus rituximab significantly prolonged PFS compared with R-CHOP in patients with indolent NHL (median PFS 69.5 vs. 31.2 months) (26). However, we acknowledge that treatment allocation in our study was not randomized: bendamustine was preferentially used for patients with hyperleukocytosis or splenomegaly, whereas CHOP/CDOP or CVP were selected for those with bulky nodal disease (mass ≥7 cm) or generalized lymphadenopathy. Specifically, bulky or extensively disseminated nodal disease has been consistently associated with adverse outcomes in indolent lymphomas, independent of treatment modality. Therefore, these findings should be interpreted with caution and require validation in prospective, randomized studies with standardized treatment allocation.

Moreover, our subgroup analyses revealed differential yet clinically meaningful patterns of obinutuzumab benefit by histology. In FL, obinutuzumab conferred significant PFS improvement (3-year PFS: 82.0% vs. 54.0%, p=0.0051), consistent with GALLIUM’s primary finding, though OS remained comparable between arms (98.5% vs. 89.8%, p=0.1). By contrast, the MZL subgroup demonstrated a pronounced and unexpected OS advantage (3-year OS: 100% vs. 82.4%, p=0.015) that was entirely driven by SMZL (100% vs. 71.0%, p=0.031), with no significant differences observed in MALT or NMZL. This MZL finding appears to diverge from GALLIUM, which reported no significant PFS or OS difference in its MZL subgroup and concluded against obinutuzumab in MZL (7). Several distinctions may account for this discrepancy. Firstly, our strict GELF-based high tumor burden criteria enriched for higher-risk patients who may derive disproportionate benefit. Secondly, GALLIUM reported MZL outcomes only in aggregate, potentially diluting a SMZL-specific signal. Thirdly, as a real-world study, our cohort reflects less selected clinical practice. While the modest SMZL sample size (n=26) warrants caution, the magnitude of the OS effect and its biological plausibility provide compelling hypothesis-generating evidence, underscoring the innovative value of precision risk stratification in high-tumor-burden MZL.

In terms of safety, FL 1-3a and MZL have a relatively low risk of TLS due to their low proliferative rate. However, the introduction of targeted drugs that induce rapid tumor reduction has increased the risk of TLS in patients with high tumor burden (27). TLS most commonly occurs during the first treatment cycle, and biological agents such as anti-CD20 monoclonal antibodies have also been shown to induce TLS. Additionally, the incidence of IRRs in patients treated with obinutuzumab was relatively high. In the phase 1b GALTON trial, which investigated the safety and preliminary efficacy of obinutuzumab combined with chemotherapy in 41 patients with CLL, IRRs were the most common AEs, occurring in 88% of patients (28).

In the current study, the treatment regimen was generally well-tolerated. All 186 patients received chemotherapy pretreatment during the first treatment cycle. No statistically significant difference was observed in the incidence of adverse events between the two groups. The most common AE associated with obinutuzumab was IRRs (grade 1-2). Additionally, TLS was observed in only 1 patient in the obinutuzumab-based group. Notably, no grade 3 or 4 IRRs occurred in our cohort, indicating that chemotherapy pretreatment can reduce the severity of IRRs. Moreover, no AEs leading to treatment discontinuation or death were observed in patients treated with obinutuzumab combined with chemotherapy in this analysis.

## 5 Conclusions

This real-world study demonstrates that obinutuzumab-based chemotherapy is associated with superior efficacy outcomes compared with rituximab-based regimens in patients with high tumor burden FL 1-3a and MZL, with a comparable safety profile. These findings support the preferential use of obinutuzumab in this challenging patient population and warrant confirmation in prospective, randomized studies specifically targeting high-risk subgroups.

## Data Availability

The original contributions presented in the study are included in the article/supplementary material. further inquiries can be directed to the corresponding author.
